# Pivotal Role of Carbohydrate Sulfotransferase 15 in Fibrosis and Mucosal Healing in Mouse Colitis

**DOI:** 10.1371/journal.pone.0158967

**Published:** 2016-07-13

**Authors:** Kenji Suzuki, Somasundaram Arumugam, Junji Yokoyama, Yusuke Kawauchi, Yutaka Honda, Hiroki Sato, Yutaka Aoyagi, Shuji Terai, Kazuichi Okazaki, Yasuo Suzuki, Shuji Mizumoto, Kazuyuki Sugahara, Raja Atreya, Markus F. Neurath, Kenichi Watanabe, Taishi Hashiguchi, Hiroyuki Yoneyama, Hitoshi Asakura

**Affiliations:** 1 Department of Gastroenterology, Niigata University Medical and Dental Hospital, Niigata city, Niigata, Japan; 2 Department of Clinical Pharmacology, Niigata University of Pharmacy and Applied Life Science, Niigata city, Niigata, Japan; 3 The Third Department of Internal Medicine, Division of Gastroenterology and Hepatology, Kansai Medical University, Moriguchi city, Osaka, Japan; 4 Internal Medicine, Toho University, Sakura Medical Center, Sakura city, Chiba, Japan; 5 Department of Pathobiochemistry, Faculty of Pharmacy, Meijo University, Nagoya city, Aichi, Japan; 6 Laboratory of Proteoglycan Signaling and Therapeutics, Graduate School of Life Science, Hokkaido University, Sapporo city, Hokkaido, Japan; 7 Department of Medicine 1, University of Erlangen-Nürnberg, Erlangen, Bavaria, Germany; 8 Stelic Institute & Co., Inc., Minato City, Tokyo, Japan; Charité-Universitätsmedizin Berlin, GERMANY

## Abstract

Induction of mucosal healing (MH) is an important treatment goal in inflammatory bowel disease (IBD). Although the molecular mechanisms underlying MH in IBD is not fully explored, local fibrosis would contribute to interfere mucosal repair. Carbohydrate sulfotransferase 15 (CHST15), which catalyzes sulfation of chondroitin sulfate to produce rare E-disaccharide units, is a novel mediator to create local fibrosis. Here we have used siRNA-based approach of silencing CHST15 in dextran sulfate sodium (DSS) induced colitis in mice, human colon fibroblasts and cancer cell lines. In a DSS-induced acute colitis model, CHST15 siRNA reduced CHST15 mRNA in the colon, serum IL-6, disease activity index (DAI) and accumulation of F4/80^+^ macrophages and ER-TR7^+^ fibroblasts, while increased Ki-67^+^ epithelial cells. In DSS-induced chronic colitis models, CHST15 siRNA reduced CHST15 mRNA in the colon, DAI, alpha-smooth muscle actin^+^ fibroblasts and collagen deposition, while enhanced MH as evidenced by reduced histological and endoscopic scores. We also found that endoscopic submucosal injection achieved effective pancolonic delivery of CHST15 siRNA in mice. In human CCD-18 Co cells, CHST15 siRNA inhibited the expression of CHST15 mRNA and selectively reduced E-units, a specific product biosynthesized by CHST15, in the culture supernatant. CHST15 siRNA significantly suppressed vimentin in both TGF-ß-stimulated CCD18-Co cells and HCT116 cells while up-regulated BMP7 and E-cadherin in HCT116 cells. The present study demonstrated that blockade CHST15 represses colonic fibrosis and enhances MH partly though reversing EMT pathway, illustrating a novel therapeutic opportunity to refractory and fibrotic lesions in IBD.

## Introduction

Inflammatory bowel disease (IBD) is a chronic, progressive, destructive disease of the gastrointestinal tract characterized by the presence of extensive ulceration and mucosal inflammation in the gut [1,2]. Treatment goals of IBD are being conceptualized and include not only symptom control alone but also prevention of structural bowel damage [3,4]. In this respect, mucosal healing (MH) judged by endoscope is now the most important parameter to evaluate the therapeutic efficacy in IBD [5,6]. MH induction-effects by the current systemic drugs are not always satisfied, for example, anti-tumor necrosis factor (TNF) agents alone can achieve only 30.1% MH in Crohn’s disease (CD) and approximately 60% MH at week 8 in ulcerative colitis (UC), in spite of their primary usage [5–7].

Although the mechanism underlying the persistence of drug-resistant or -recurrent active mucosal lesions is unexplored, additional local factors that are not fully controlled by systemic anti-inflammatory and/or immune-modulating drugs alone may exist. We hypothesized that local fibrosis is an additional candidate that interferes MH, since refractory ulcers often associte with fibrosis not only in drug-resistant peptic ulcer and esophageal ulcer post submucosal dissection but also in IBD [8–12]. Fibrosis involves multi-factorial cascades including up-regulation of profibrotic factors, imbalances of matrix metalloproteinase (MMP)-tissue inhibitor of metalloproteinase (TIMP), epithelial mesenchymal transition (EMT)-mesenchymal epithelial transition (MET), extracellular matrix (ECM) production-dissolution and tissue repair-remodeling [8–10]. Regulation of these imbalances is still a challenge, for example, blockade of a profibrotic factor, transforming growth factor (TGF)-ß, reduces fibrosis but might inhibit regulatory T cell functions leading to severe inflammation, whilst promotion of regulatory T cell immunity reduces inflammation but might therefore augment the risk of fibrosis and additionally, cancer through increased activity of TGF-ß.

Carbohydrate sulfotransferase 15 (CHST15), formerly known as *N*-acetylgalactosamine 4-*O*-sulfate 6-*O*-sulfotransferase (GalNAc4S-6ST), is a putative type II transmembrane Golgi protein involved in the biosynthesis of highly sulfated disaccharide units (E-units) of chondroitin sulfate (CS), which binds to various proinflammatory and profibrotic mediators, adhesion molecules, receptor for advanced glycation end-product (RAGE) and pathogenic microorganisms [13–16]. All of these are involved in fibrogenesis and therefore characterize CHST15 and/or CS-E axis as a “fine-tuner” of local fibrosis. We have recently shown that blockade of CHST15 by siRNA repressed cardiac fibrosis in rats with dilated cardiomyopathy through suppressing multiple mediators associated with cardiac remodeling such as interleukin (IL)-1ß, IL-6, monocyte chemoattractant protein (MCP)-1/CCL2, collagen type 1 and 3, MMP-2, MMP-9, connective tissue growth factor (CTGF), TGF-ß and TGF-ß activated kinase (TAK)-1 [17].

To investigate whether CHST15 is also involed in intestinal fibrosis and its impact on MH, we conducted functional blocking experiments using CHST15 siRNA in the present study. CHST15 siRNA repressed intestinal fibrosis in mouse colitis surprisingly without exacerbating ulcerative lesion. Rather, CHST15 siRNA enhanced MH indicating that blockade of CHST15 skews host responses from fibrotic healing towards adequate tissue repair. A mechanism on this skewness is attributable in part to EMT-reversing machinery of CHST15 siRNA based on *in vitro* colon fibroblast experiments.

## Materials and Methods

All animal experiments complied with the guidelines set by the Niigata University School of Medicine and Stelic Institute & Co., Inc. Mouse model of colitis—The protocol was approved by Stelic IACUC (approval number: RP-51, 52, 53, 54, 55). All animals were housed and cared for in accordance with the Japanese Pharmacological Society Guidelines for Animal Use. The animals were sacrificed by exsanguination through direct cardiac puncture under ether anesthesia.

### Experimental mouse colitis and endoscopy

**Fig 1:**
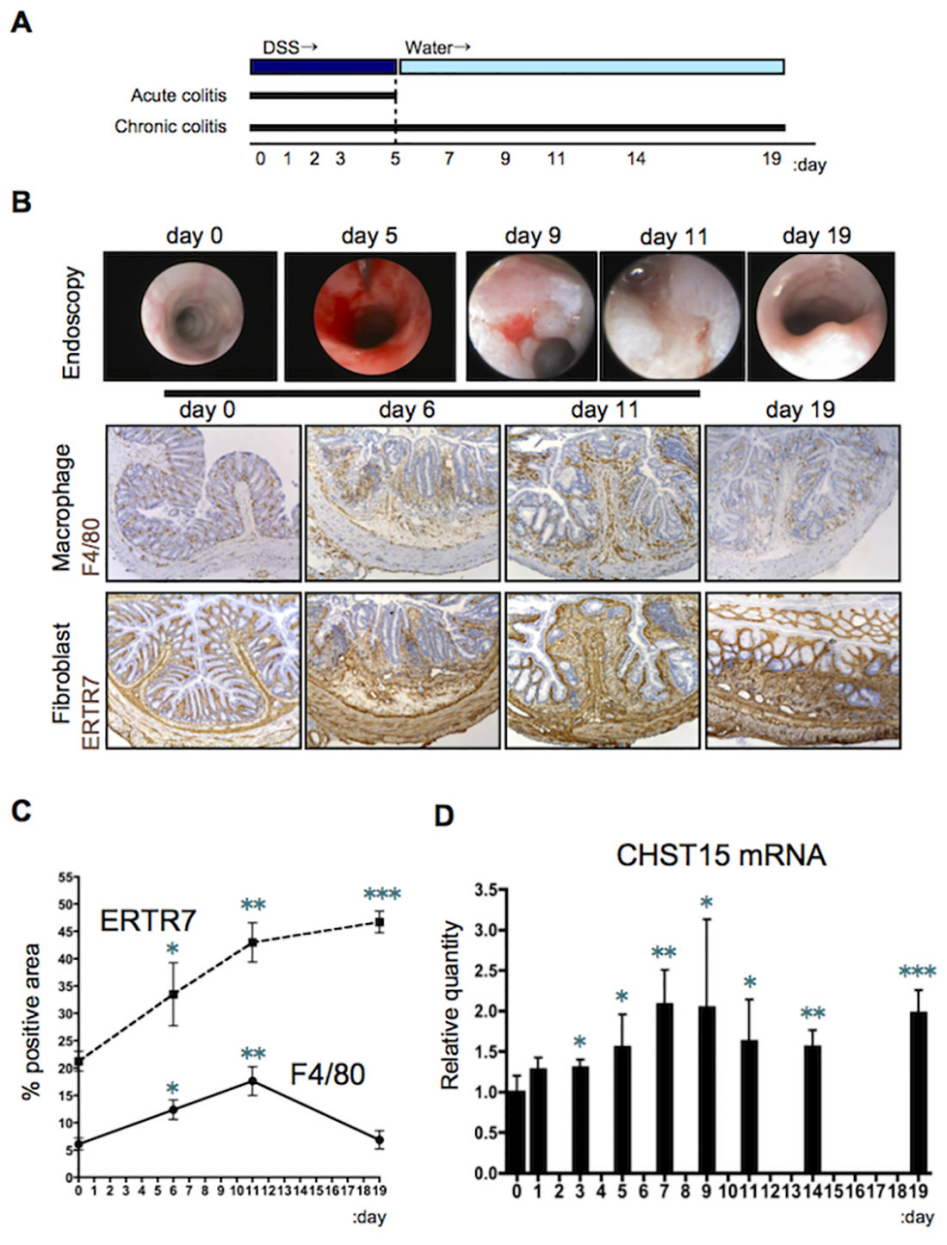
Expression of CHST15 mRNA during the shift from inflammation to fibrosis in mouse colitis. **(A)** Schematic representation of acute and chronic DSS colitis. (**B**) Endoscopic and histological findings. Representative photos of immunostaining for F4/80 and ER-TR7 (brown) in mice of disease controls (without performing siRNA injection) at indicated times are shown. Original magnification, x200. (**C**) Kinetics of positive area (%) for ER-TR7 (dotted line) and F4/80 (black line) of disease control mice. (**D**) Kinetics of CHST15 mRNA expressions in the colon of disease control mice. Results are expressed as mean ± SD (n = 4~6). *p<0.05, **p<0.01 and ***p<0.001 vs. normal mice (day 0) by Student’s t-test. Representative data were shown from 3 independent experiments.

**Fig 2:**
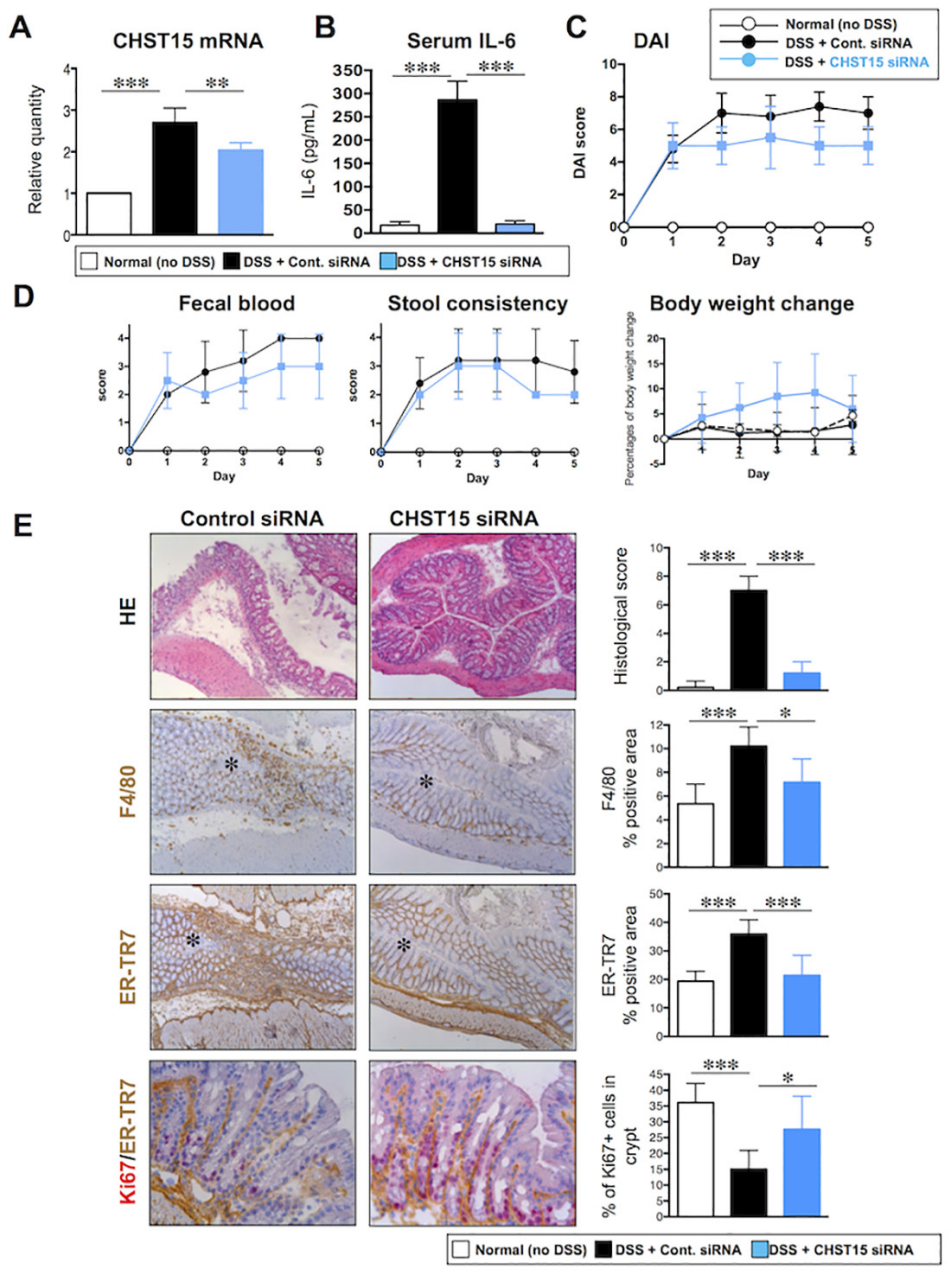
Enhanced MH by systemic CHST15 siRNA in mouse acute colitis. (**A**) Effect of CHST15 siRNA on the expressions of CHST15 mRNAs at day 5. CHST15 siRNA or negative control siRNA was injected intraperitoneally (i.p.) 2 days after DSS and mice were sacrificed at day 5. Statistical analyses using one-way ANOVA with Bonferroni multiple comparison test are shown; normal (no DSS; white bar) vs. negative control siRNA (DSS + control siRNA; black bar), negative control siRNA vs. CHST15 siRNA (DSS + CHST15 siRNA; blue bar). (**B**) Effect of CHST15 siRNA on serum IL-6 level at day 5. (**C**) Disease activity index (DAI). P<0.001; negative control siRNA vs. CHST15 siRNA by two-way ANOVA with Bonferroni multiple comparison test. (**D**) 3 components (fecal blood score, stool consistency score and % reduction of body weight). P<0.05 (in fecal blood score and body weight change); negative control siRNA vs. CHST15 siRNA by two-way ANOVA with Bonferroni multiple comparison test. (**E**) Left panels: Representative HE and immunostaining for ER-TR7 (brown), F4/80 (brown) and Ki-67 (red, double stained with ER-TR7) of the colon at day 5. Original magnifications, x100 for single staining and x400 for double staining. Asterisk indicates the lumen. Right panels: Effect of CHST15 siRNA on histological score, % of F4/80^+^ cells, % of ER-TR7^+^ cells and % of Ki-67^+^ cells in crypt at day 5. Results are expressed as mean ± SD (n = 3~6). *p<0.05, **p<0.01 and ***p<0.001 vs. negative control siRNA treatment group by one-way ANOVA with Bonferroni multiple comparison test. Representative data were shown from 3 independent experiments.

**Fig 3:**
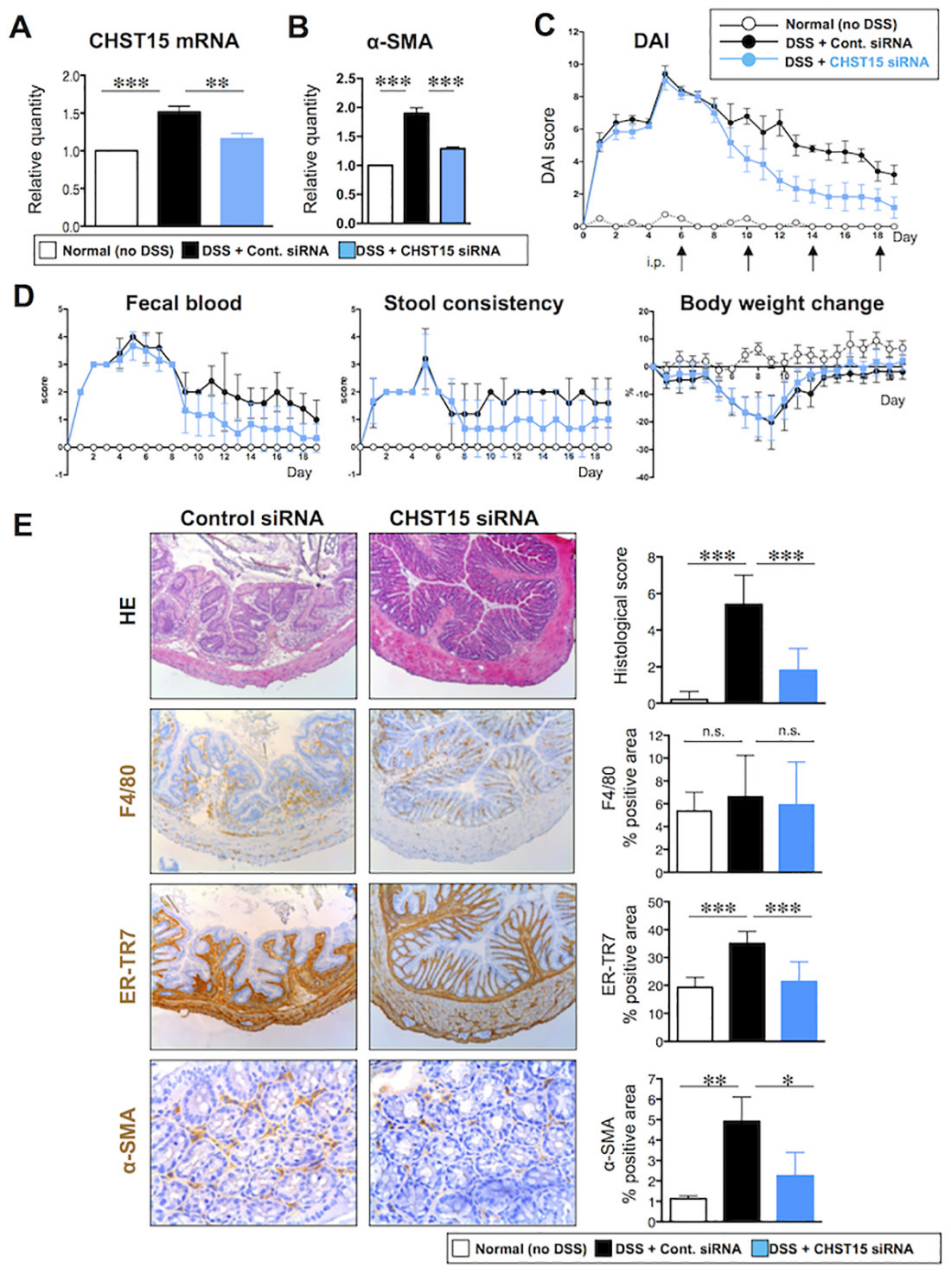
Repression of intestinal fibrosis by systemic CHST15 siRNA in mouse chronic colitis. (**A**) Effect of CHST15 siRNA on the expressions of CHST15 mRNAs at day 19. CHST15 siRNA or negative control siRNA was injected intraperitoneally (i.p.) for 4 times (days 6, 19, 14, 18) and mice were sacrificed at day 19. Statistical analyses using one-way ANOVA with Bonferroni multiple comparison test are shown; normal (no DSS; white bar) vs. negative control siRNA (DSS + control siRNA; black bar), negative control siRNA vs. CHST15 siRNA (DSS + CHST15 siRNA; blue bar). (**B**) Effect of CHST15 siRNA on the expression of α-SMA mRNA at day 19. (**C**) Disease activity index (DAI). P<0.001; negative control siRNA vs. CHST15 siRNA by two-way ANOVA with Bonferroni multiple comparison test. (**D**) 3 components (fecal blood score, stool consistency score and % reduction of body weight). P<0.001 (in fecal blood score and stool consistency score) and p<0.01 (in % reduction of body weight); negative control siRNA vs. CHST15 siRNA by two-way ANOVA with Bonferroni multiple comparison test. (**E**) Left panels: Representative HE and immunostaining for ER-TR7 (brown), F4/80 (brown) and α-SMA (brown) of the colon at day 19. Original magnifications, x100 except for α-SMA (x400). Right panels: Effect of CHST15 siRNA and negative control siRNA on histological score, % of F4/80^+^ cells, % of ER-TR7^+^ cells and % of α-SMA^+^ cells at day 19. Results are expressed as mean ± SD (n = 3~6). *p<0.05, **p<0.01 and ***p<0.001 vs. negative control siRNA treatment group by one-way ANOVA with Bonferroni multiple comparison test. Representative data were shown from 3 independent experiments.

Specific pathogen-free female C57BL/6 mice (7–9 weeks old) were obtained from CLEA Japan Inc. Colitis was induced in mice by administering 5% DSS (MW: 5,000; Wako) or 3% DSS (MW 36,000–50,000; MP Biomedicals) in distilled water *ad libitum* for 5 days [18–20]. For chronic colitis, DSS was switched to regular drinking water from day 5 to day 19 (Fig 1) [20]. The disease activity index (DAI) was determined as a combination of body weight, stool consistency and fecal blood as described previously [18–20]. For blocking experiments via systemic route, CHST15 siRNA [21] or negative control siRNA (“Control”) [21] was diluted with 0.1% (w/v) atelocollagen (KOKEN)/ phosphate-buffered saline (PBS). The following sense and anti-sense sequences were used as a negative control: 5’-AUCCGCGCGAUAGUACGUA-TT-3’ (sense), 5’-TACGTACTATCGCGCGGAT-TT-3’ (antisense). One μg/200 μL of siRNA-atelocollagen complex was administered intraperitoneally (i.p.) per mouse at day 2 after DSS administration for acute colitis (Fig 2) and days 6, 10, 14, 18 for chronic colitis (Fig 3). For blocking experiments via submucosal route, CHST15 siRNA or negative control siRNA was injected submucosally (s.m.) at once via mouse endoscopy system (Karl-Storz) on day 6. One μg of CHST15 siRNA or negative control siRNA did not induce serum IL-6 and IFN-γ levels, or did not show any evidence of histological inflammation when injected into normal mouse, rat and cynomolgus monkey (data not shown). Segmental simple endoscopic score for CD (SES-CD) was estimated in a rectal segment as a combination of following parameters; size of ulcer (0–3), ulcerated surface (0–3), other affected surface (0–3) and narrowing (0–3) [22]. Serum IL-6 concentration was determined by mouse IL-6 Quantikine ELISA kit (R&D systems). Colon hydroxyproline content was measured as previously described [23]. Protein concentrations of colon samples were determined using BCA protein assay kit (Thermo Fisher Scientific) and used to normalize the calculated hydroxyproline values. Colon hydroxyproline levels were expressed as mg per μg protein. Additional experiments were performed to identify the effect of CHST15 siRNA on a severe acute colitis model using compound 48/80 and DSS (S1 File). All animal experiments complied with the guidelines set by the Niigata University School of Medicine, Niigata, Japan and Stelic Institute & Co., Inc., Tokyo, Japan.

### Gene expression analysis

Total RNA was extracted from the colon using RNAiso (TaKaRa, Japan) and reverse transcribed [20,24,25]. Thereafter cDNA was amplified using real-time PCR DICE and SYBR premix Taq (TaKaRa, Japan) with a set of primers corresponding to CHST15, alpha-smooth muscle actin (α-SMA), ß-actin or glyceraldehyde-3-phosphate dehydrogenase (GAPDH) as previously described [20,24,25].

### Histochemistry

The distal colon was sampled and fixed in 4% formalin, embedded in paraffin and stained with hematoxylin and eosin (HE) or sirius red as per previous methods [18,24,25]. Immunohistochemical staining was performed as reported earlier [25]. The following anti-mouse Abs were used: F4/80 (Caltag), ER-TR7 (BMA), Ki-67 (COSMO-Bio), CS56 (Seikagaku) and α-SMA (Abcam). Anti-mouse monoclonal Ab to CHST15 was made in house. Histological score was measured by summation of the following parameters [18]. Epithelium score was as follows; 0 = normal morphology; 1 = loss of goblet cells; 2 = loss of goblet cells in large areas; 3 = loss of crypts; and 4 = loss of crypts in large areas. Infiltrate score was as follows; 0 = no infiltrate; 1 = infiltrate around crypt basis; 2 = infiltrate reaching the L. muscularis mucosae; 3 = extensive infiltration reaching the L. muscularis mucosae and thickening of the mucosa with abundant edema; and 4 = infiltration of the L. submucosa. For quantification of immunostained and sirius-red stained areas, bright field images were captured using a digital camera (DFC280, Leica Microsystems) at 200-fold magnification and the positive areas in 5 fields/section were measured using ImageJ software (National Institute of Health) [24]. The area of Ki-67^+^ cells was measured in 10 crypts per each section.

### Visualization of submocosally injected siRNA in mice

Thirty minutes after submucosal injection of carboxyfluorescein (FAM)-labeled CHST15 siRNA, normal mice were sacrificed and the colon sections were examined by differential interference contrast microscopy (Leica Microsystems). In separate experiments, the whole colons were examined by streoscopic microscopy (Leica Microsystems).

### ELISA to detect CHST15 siRNA

ELISA was performed to detect siRNA in the colon of normal mouse according to Yu’s method [26]. Colons were extracted at 4, 24, 48 and 144 hours after submucosal injection of CHST15 siRNA. In brief, total RNA of colon sample was transferred to 5% DMSO-PBS in a PCR tube and incubated at 80°C. The 3’-biotinylated template probe (Japan Bio Servies) solution was added to the sample solution and incubated. After hybridization, the sample solution was incubated in a streptavidin-coated 96-well plate. After washing, each well was incubated with the digoxigenin-labeled ligation probe (Japan Bio Servies) solution containing T4 DNA ligase (TaKaRa). Wells were washed with the washing buffer, with deionized water, and then incubated with the peroxidase-labeled anti-digoxigenin (1:1,000 dilution, Roche, Switzerland). After washing, enzymatic activity was detected using tetramethylbenzidine (TMB) (Sigma-Aldrich) and the absorbance was measured at 450 nm. To construct a standard curve, standards were prepared with known concentrations of CHST15 siRNA. The concentration of CHST15 siRNA in the colon was calculated from the standard curve.

### *In vitro* silencing experiments

CCD-18Co cells (human colon cell line) were purchased form ATCC (USA). The concentrations of CHST15 siRNA and negative control siRNA used were 5 pM, 50 pM, 500 pM, 5 nM and 50 nM. Five hundred μL/well of Opti-MEMl (GIBCO), siRNA and 7.5 μL/well of RNAiMAX-Reagent (Invitrogen) were incubated on a 6-well plate at room temperature for 20 minutes. CCD-18Co cells (250,000 cells) were suspended in 2.5 mL of the basic culture medium in a CO_2_ incubator for 48 hours (2.5 mL/well). Culture supernatant was tested for IL-6 concentration (R&D systems). Total RNA was extracted from each transfected cell using a FastPure RNA kit (TaKaRa, Japan) according to the manufacturer’s instructions. Further, cDNA was synthesized and real-time RT-PCR was performed using SYBR premix Taq (TaKaRa). The expression of the human CHST15 gene was normalized by the expressed amount of RNA of human GAPDH. In separate series of experiments using CCD18-Co cells and human colon cancer cell line (HCT116) cells, the above experiments were performed in the presence of recombinant TGF-ß1 (10 ng/mL) for 48 h.

### Extraction of glycosaminoglycans and disaccharide composition analysis

Conditioned medium from siRNA-treated CCD-18Co cells were treated with 80% ethanol containing 1% sodium acetate at 4°C overnight. The resultant precipitate was reconstituted in water, boiled for 10 min and digested with actinase E as described previously [27]. The digestion was carried out at 60°C overnight. Following incubation, the sample was precipitated with 5% trichloroacetic acid and centrifuged. The supernatant was treated with diethylether to remove trichloroacetic acid. The aqueous phase was neutralized using 1 M sodium carbonate, and treated with 80% ethanol containing 1% sodium acetate at 4°C overnight. The precipitated crude glycosaminoglycan fraction was recovered by centrifugation.

The disaccharide composition of the CS from the conditioned medium from siRNA-treated CCD-18Co cells was determined as previously described [27]. Briefly, the sample was dissolved in water, and an aliquot was digested with a mixture of chondroitinases ABC and AC-II, the digest was labeled with 2-aminobenzamide to increase the sensitivity of the detection of disaccharide products, and excess 2-aminobenzamide was removed by extraction with chloroform. The fluorophore-labeled digests were analyzed by anion-exchange HPLC on a PA-03 silica column (YMC Co., Kyoto, Japan). Identification and quantification of the resulting disaccharides were achieved by comparison with the elution positions of CS-derived authentic unsaturated disaccharides.

### Data analysis and statistical methods

Statisfical analyses were performed using one-way ANOVA with Bonferroni Multiple Comparison Test on GraphPad PRISM6 (Prisim software, USA). P values < 0.05 were considered statistically significant. Results were expressed as mean ±SD. DAI and its components were analysed using two-way ANOVA with Bonferroni Multiple Comparison Test. A trend or tendency was assumed when a one-tailed Student’s t-test returned P values <0.10. P values < 0.05 were noted as # in the figure.

## Results

### Inducible expression of CHST15 in a murine model of colitis

To investigate the role of CHST15 in intestinal fibrosis, we characterized the kinetics of CHST15 mRNA expression in the colon during acute and chronic experimental colitis in mice. Acute colitis with mucosal injury was induced by DSS in drinking water for 5 days (Fig 1A). Chronic colitis with intestinal fibrosis was induced by treatment with DSS in drinking water for 5 days and followed by normal water for 2 weeks (Fig 1A) [20]. High-resolution endoscopy of the colon showed massive hemorrhage at day 5, ulcerative lesions at day 9, progression to ulcer-based fibrosis at day 11 and narrowing of the lumen at day 19 (Fig 1B). Histologic analyses revealed increased numbers of F4/80^+^ macrophages and ER-TR7^+^ fibroblasts associated with submucosal edema at day 6 compared to the findings at day 0 (Fig 1B). The area of F4/80^+^ macrophages showed a peak at day 11 and subsequently decreased over time, while the area of ER-TR7^+^ fibroblasts increased until day 19 (Fig 1C). CHST15 mRNA expression increased significantly from day 3, reached a peak around days 7 to 9 and slightly decreased thereafter (Fig 1D). Taken together with the endoscopic and histological findings, these data suggested that CHST15 mRNA is inducible in response to tissue damage and shows high levels of expression when endoscopically active ulcers accompanied by marked accumulation of macrophages and fibroblasts are evident (day 9).

### Enhanced epithelial renewal by CHST15-silencing in a murine model of acute colitis

Although over accumulation of activated fibroblasts contributes to local fibrotic lesion development, accumulation of adequate grade of fibroblasts might contribute to reseal the damaged epithelium. Thus, we initially tested whether CHST15 blockade induces delayed epithelial healing in a mouse model of acute colitis (Fig 1A). Two days following DSS treatment, the atelocollagen complex with CHST15 siRNA or negative control siRNA was administered intraperitoneally. At day 5, the expression level of CHST15 mRNA was increased in the colon of DSS + negative control siRNA-treated mice compared to normal mice (Fig 2A). In contrast, CHST15 siRNA significantly reduced the expression of CHST15 mRNA compared to negative control siRNA (Fig 2A). CHST15 siRNA also reduced serum levels of IL-6, an established marker of inflammation in this model (Fig 2B). DAI at days 4 and 5 significantly decreased by CHST15 siRNA (Fig 2C) and this was mainly attributable to reduced fecal blood score from an early time point (day 3) (Fig 2D). Focal accumulation of F4/80^+^ inflammatory macrophages and ER-TR7^+^ fibroblasts was observed at day 5 and this was inhibited by CHST15 siRNA treatment almost to a normal level (Figs 1C and 2E). Massive mucosal defects were shown in negative control siRNA, while in contrast, epithelial architecture was well preserved by CHST15 siRNA treatment (Fig 2E). The number of Ki-67^+^ epithelial cells was significantly increased in CHST15 siRNA-treated mice compared to negative control (Fig 2E), indicating that CHST15 siRNA promoted epithelial renewal.

### Repression of fibrosis by CHST15-silencing in a murine model of chronic colitis

To evaluate the *in vivo* function of CHST15 in chronic colitis, an atelocollagen complex with CHST15 siRNA or negative control siRNA was administered intraperitoneally at days 6, 10, 14 and 18. At day 19, the expression of CHST15 mRNA showed still high level in the colon of DSS + negative control siRNA-treated mice compared to normal mice (Figs 1A and 3A). A significant reduction of CHST15 mRNA expression was observed at day 19 upon CHST15 siRNA treatment as compared to negative control siRNA treatment, suggesting successful targeting of CHST15 *in vivo* (Fig 3A). CHST15 siRNA also reduced the expression of α-SMA mRNA, an activation marker of fibroblasts in this model (Fig 3B). A significant reduction of DAI in the CHST15 siRNA was observed during the course of fibrosis progression (Fig 3C), and again, this was mainly attributable to reduced fecal blood score from an early time point, day 10 (Fig 3D). Fibrosis, edema and inflammation were reduced, and the epithelial architecture was preserved by CHST15 siRNA treatment compared to negative control siRNA (Fig 3E). Extensive accumulations of ER-TR7^+^ fibroblasts and α-SMA^+^ activated myofibroblasts were observed at day 19 and these were inhibited by CHST15 siRNA treatment almost to a normal level (Figs 1C and 3E). In contrast, CHST15 siRNA did not affect the area of F4/80^+^ macrophages at day 19 (Fig 3E).

### Submucosal injection for pancolonic delivery of CHST15 siRNA in mice

**Fig 4:**
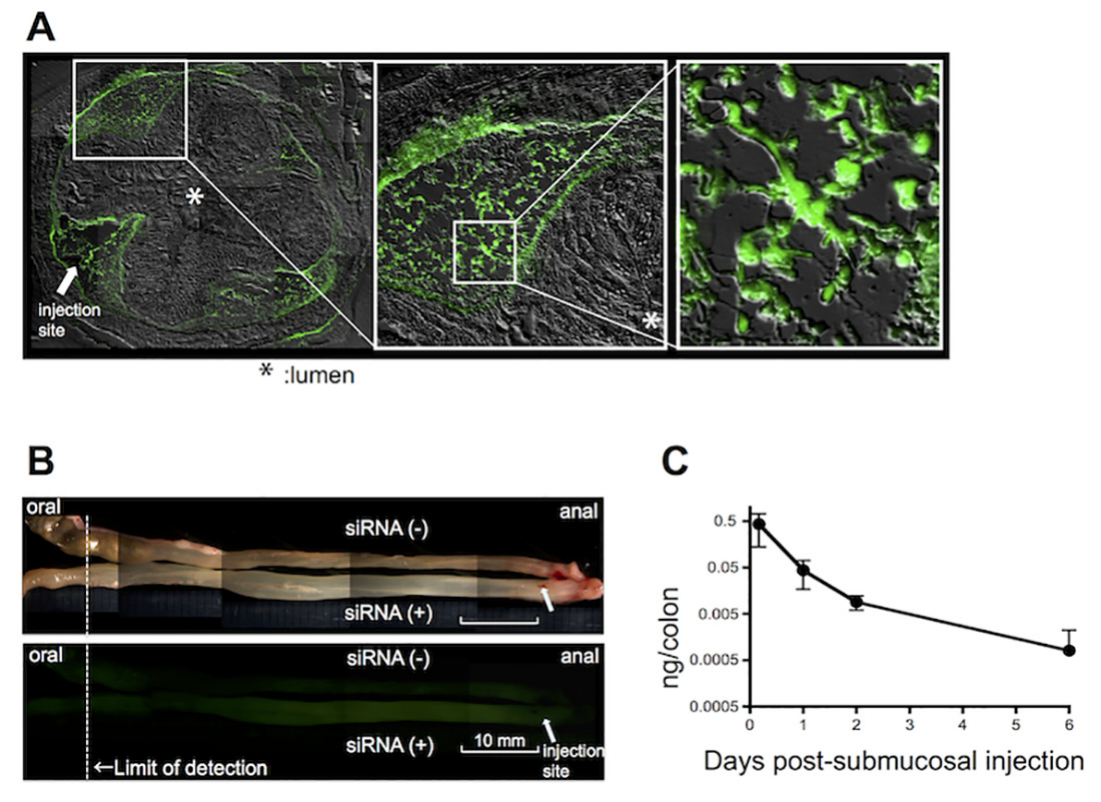
Submucosal injection as a pancolonic delivery of siRNA in mouse colon. (**A**) Retention of FAM-labeled CHST15 siRNA within the submucosa of the normal mouse colon by differential interference contrast microscopy. Arrow indicated the site of injection. FAM-positive cells were clearly detected at higher magnifications (open squares). Original magnification, x100. (**B**) Diffusion of FAM-labeled siRNA in the whole normal mouse colon by stereoscopic microscopy. Lower panel showed fluorescence signals. Arrow indicated the site of injection. Non-injected colon was placed as a negative control. Original magnification, x7.3. (**C**) Concentrations of CHST15 siRNA in the normal mouse colon 4, 24, 48 and 144 h after a single submucosal injection.

To translate the therapeutic potential of oligonucleotide-based medicine into clinical practice, it is important to increase targeting and retention of siRNA at sites of injury. We took an approach utilizing submucosal injection, which may allow siRNA to retain locally and release it to locally affect surrounding cells. First, we visualized the distribution of FAM-labeled CHST15 siRNA after submucosal injection in the normal mouse rectum. FAM-labeled siRNA was detected not only at the injected site but also at the opposite site within the submucosa (Fig 4A). Cells having contact with matrix fiber in the submucosa showed uptake of FAM-labeled siRNA (Fig 4A), suggesting successful incorporation in cells. Stereoscopic observation revealed that the fluorescence signal was detectable approximately 6 cm far from the site of injection, although the signal was gradually decreased (Fig 4B). Thus, injected siRNA can rapidly spread in a circumferential, pancolonic fashion within the submucosa from the rectum to the cecum in mice. Next we established an ELISA system to detect the concentration of CHST15 siRNA in mouse colon. CHST15 siRNA was detectable from 4 h after submucosal injection and decreased thereafter but was still detectable at day 6 after injection (Fig 4C). Since the ELISA system detects free, unbound siRNA, these results suggested that injected siRNA could be retained within matrix space and incorporated into cells in a sustained manner. Thus, the submucosal space may function as a reservoir of siRNA.

### Submucosal injection for effective function of CHST15 siRNA in mouse colitis

**Fig 5:**
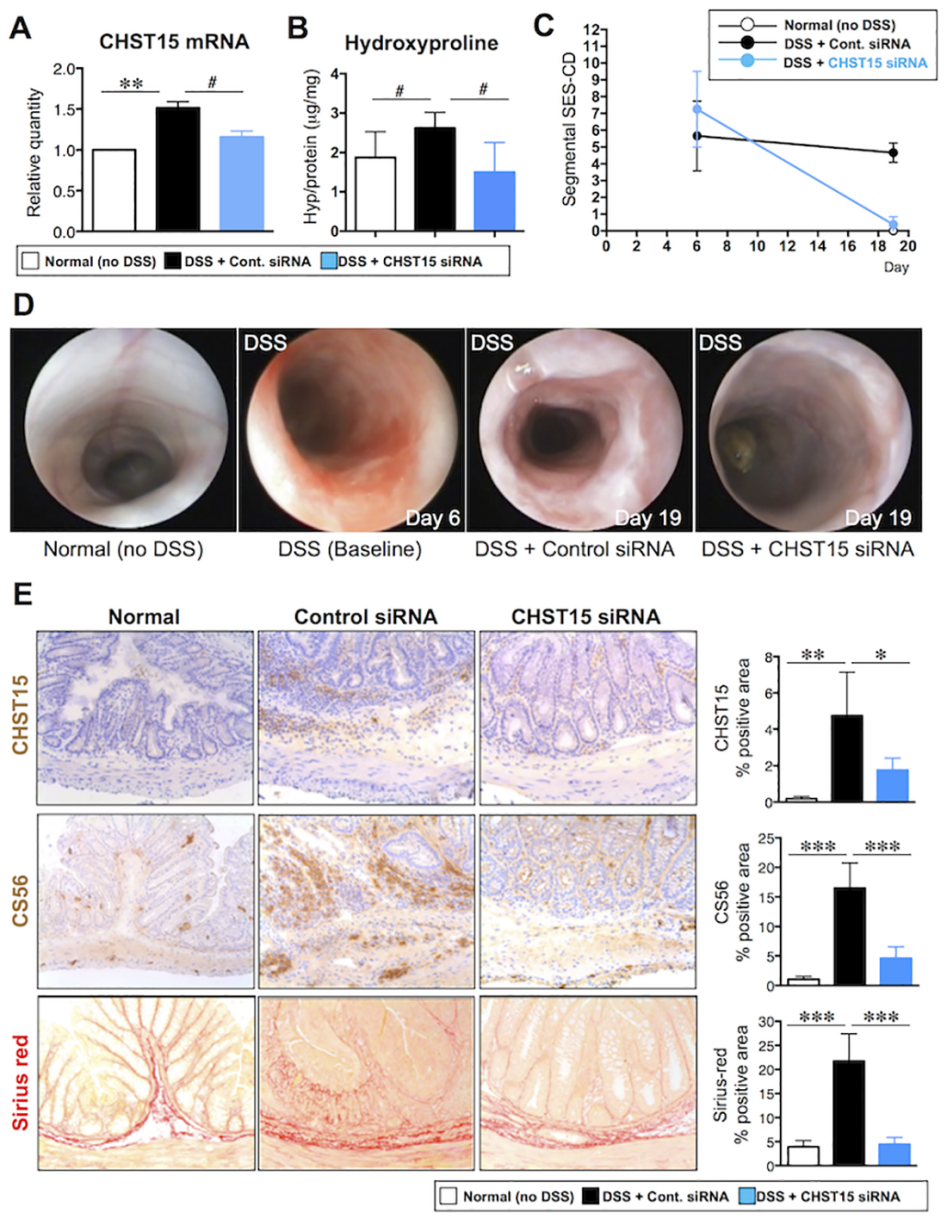
Repression of intestinal fibrosis and induction of MH by submucosally injected CHST15 siRNA in mouse chronic colitis. **(A)** Effect of CHST15 siRNA by submucosal injection on the expressions of CHST15 mRNAs at day 19. CHST15 siRNA or negative control siRNA was injected submucosally (s.m.) via endoscopy at day 6 and mice were sacrificed at day 19. Statistical analyses using one-way ANOVA with Bonferroni multiple comparison test (**) or Student’s t-test (#) are shown; normal (no DSS; white bar) vs. negative control siRNA (DSS + control siRNA; black bar), negative control siRNA vs. CHST15 siRNA (DSS + CHST15 siRNA; blue bar). (**B**) Effect of CHST15 siRNA on the colon hydroxyproline content at day 19. #: P<0.05; negative control siRNA vs. CHST15 siRNA by Student’s t-test. (**C**) Simple endoscopic score for CD (SES-CD) in rectal segment at baseline (day 6) and endpoint (day 19). P<0.001; negative control siRNA vs. CHST15 siRNA by one-way ANOVA with Bonferroni multiple comparison test. (**D**) Representative endoscopic findings in the rectum of normal (no DSS; left), DSS only as baseline (day 6, middle left), negative control siRNA at day 19 (DSS + Control siRNA; middle right) and CHST15 siRNA at day 19 (DSS + CHST15 siRNA; right). (**E**) Left panels: Representative immunostaining for CHST15 (brown), for CS with anti-CS antibody CS56 (brown) and for collagen with Sirius red (red)-staining of the colon at day 19. Original magnifications, x200. Right panels: Effect of CHST15 siRNA on % of CHST15^+^ cells, % of CS56^+^ area and % of sirius red^+^ area at day 19. Results are expressed as mean ± SD (n = 3~6). *p<0.05, **p<0.01 and ***p<0.001 vs. negative control siRNA treatment group by one-way ANOVA with Bonferroni multiple comparison test. Representative data were shown from 3 independent experiments.

Based on the pancolonic delivery, we next investigated the *in vivo* function of submucosally injected CHST15 siRNA in DSS-induced chronic colitis in mice. Naked CHST15 siRNA or negative control siRNA was injected submucosally at once 1 day after stopping DSS, when fibrotic responses including submucosal edema were already established [20]. CHST15 siRNA effectively reduced CHST15 mRNA expression at days 11 and 19 compared to negative control siRNA (Fig 5A). In order to estimate the collagen content, we have measured the hydroxyproline content of the colon samples and identified that CHST15 siRNA treatment significantly reduced the colon hydroxyproline content (Fig 5B). As efficacy endpoints, we selected endoscopic and histological analyses because these can be estimated also in clinical studies. In analogy to the simple endoscopic score for CD (SES-CD) [22] that includes 4 parameters such as size of ulcers (0 to 3), ulcerated surface (0 to 3), other affected surface (0 to 3) and narrowing (0 to 3), we estimated these parameters in rectal segments by endoscopy (Fig 5C and 5D). Compared to baseline (day 6, 1 day after stopping DSS), segmental SES-CD remained almost stable at day 19 in negative control animals (Fig 5C and 5D), suggesting persistent inflammation. In contrast, the score was significantly reduced to almost normal levels in CHST15 siRNA-treated mice at day 19 (Fig 5C and 5D). Histological analyses demonstrated that CHST15 siRNA clearly reduced CHST15 protein levels as well as CS56 levels in the colon at day 19 compared to negative control siRNA (Fig 5E), indicating that CHST15 siRNA actually inhibited the expression of CHST15 mRNA, CHST15 protein and CS56 *in vivo*. Sirius-red positive fibrosis could be detectable in a transluminal manner in control animals, while the area positive for serius-red was significantly reduced to almost normal levels by treatment with CHST15 siRNA (Fig 5E). It was noted that CHST15 siRNA-treated colons exhibited almost normal epithelial architecture including fine goblet cell morphology (Fig 5E).

### Suppression of EMT signaling pathway in human colonic fibroblasts by CHST15-silencing

**Fig 6:**
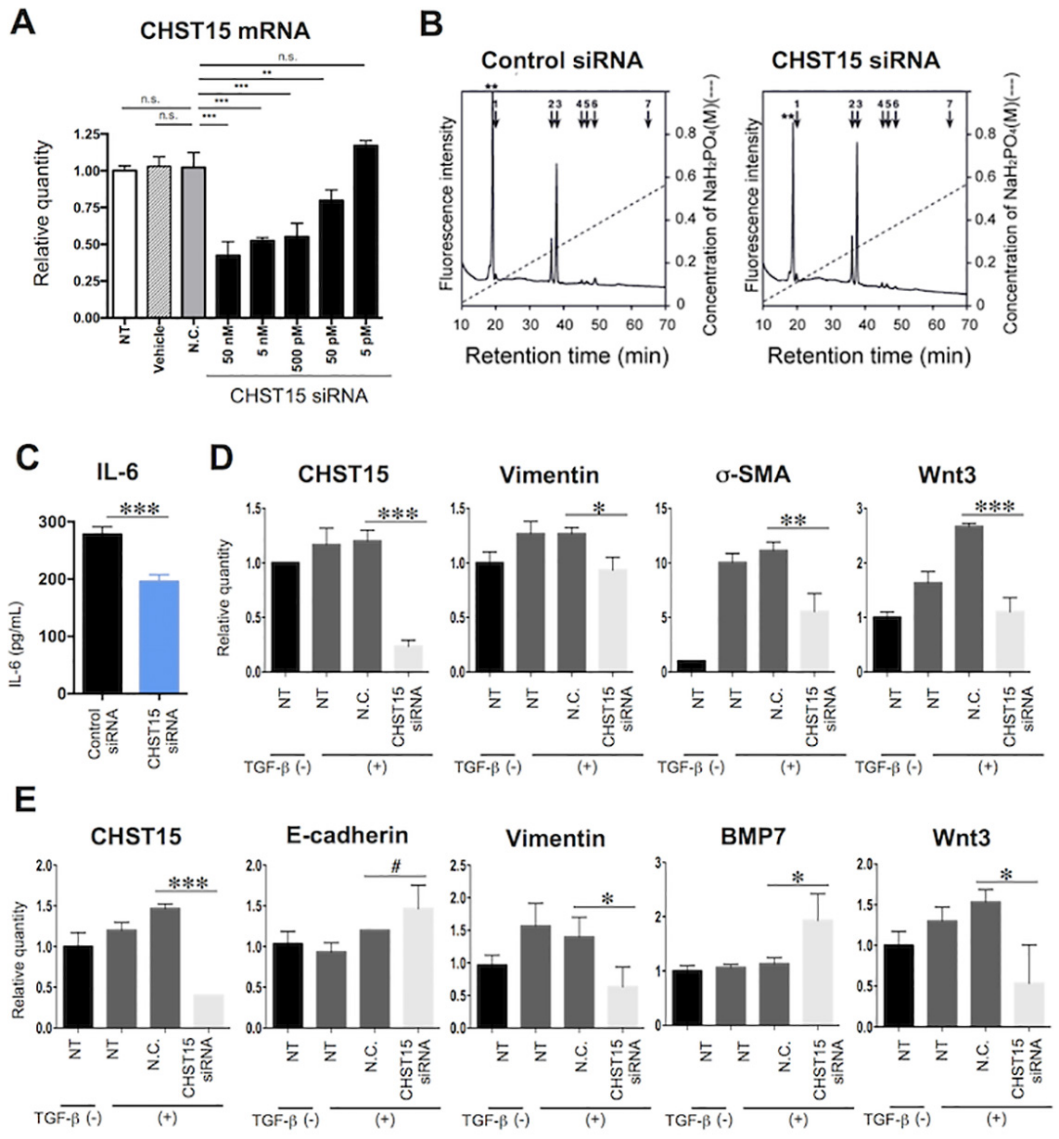
Suppression of activation pathways in human colonic fibroblasts *in vitro* by CHST15-silencing. (**A**) *In vitro* silencing efficacy in CCD-Co18 cells. Relative quantification of CHST15 mRNA by CHST15 siRNA at concentrations of 5 pM, 50 pM, 500 pM, 5 nM and 50 nM is shown. NT; non-treated cell, Vehicle; vehicle without siRNA-treated cell, N.C.; negative control siRNA-treated cell. Statistical analyses are shown; NT (white bar) vs. N.C. (gray bar), Vehicle (dashed bar) vs. N.C. (gray), N.C. vs. graded doses of CHST15 siRNA (black). (**B**) Representative anion-exchange HPLC of CS fractions in conditioned medium of control siRNA- or CHST15 siRNA-treated CCD-18Co cells. The elusion positions of authentic 2AB-labeled disaccharides are indicated by bars. 1, ΔHexUA-GalNAc; 2, ΔHexUA-GalNAc(6-*O*-sulfate); 3, ΔHexUA-GalNAc(4-*O*-sulfate); 4, ΔHexUA(2-*O*-sulfate)-GalNAc(6-*O*-sulfate); 5, ΔHexUA(2-*O*-sulfate)-GalNAc(4-*O*-sulfate); 6, ΔHexUA -GalNAc(4,6-*O*-disulfate); 7, ΔHexUA(2-*O*-sulfate)-GalNAc(4,6-*O*-disulfate). The peaks marked by *asterisks* indicate the elution positions of 2-aminobenzamide-labeled ΔHexUA-GlcNAc derived from hyaluronan. ΔHexUA, GalNAc and GlcNAc represent 4,5-unsaturated hexuronic acid, *N*-acetylgalactosamine and *N*-acetylglucosamine, respectively. (**C**) IL-6 concentration in the above (B) conditioned medium of control siRNA- or CHST15 siRNA-treated CCD-18Co cells. ***P<0.001, Student’s t-test. (**D**) Effect of CHST15 siRNA on the expression of activated markers, vimentin, α-SMA and Wnt3 mRNA by TGF-ß-stimulated fibroblasts. NT; non-treated cell, N.C.; negative control siRNA-treated cell. (**E**) Effect of CHST15 siRNA on the expression of markers related to EMT, E-cadherin, vimentin, BMP7 and Wnt3 mRNA by TGF-ß-stimulated colon cancer cell line. NT; non-treated cell, N.C.; negative control siRNA-treated cell. Results are expressed as mean ± SD (n = 3). *p<0.05, **p<0.01 and ***p<0.001 vs. negative control siRNA treatment group by one-way ANOVA with Bonferroni multiple comparison test. #p<0.05 vs. negative control siRNA treatment group by Student’s t-test.

Finally, to investigate the molecular mechanism of CHST15 siRNA in intestinal fibrosis, we selected a human colonic fibroblast cell line, CCD-18 Co, and tested the effects of CHST15 siRNA *in vitro*. CHST15 siRNA effectively reduced CHST15 mRNA in CCD-18 Co cells in a dose dependent fashion (Fig 6A). A significant silencing effect of CHST15 siRNA was observed from 50 pM concentrations. The silencing effect reached almost stable at 50 nM and even higher concentrations up to 200 nM did not cause up-regulation of IFN response-related genes, IFN-induced transmembrane protein (IFITM) or 2’,5’-oligoadenylate synthase 1 (OAS1) (data not shown). Disaccharide composition in culture supernatant was tested to estimate whether CHST15 siRNA inhibited synthesis of its product, E-units of CS. CHST15 siRNA selectively reduced the portion of E-units (Fig 6B, Table 1), indicating that CHST15 siRNA inhibits biosynthesis of E-units by colonic fibroblasts. Production of IL-6 by CCD-18 Co cells was also inhibited by CHST15 siRNA (Fig 6C), suggesting inhibited activation of colonic fibroblasts. Further, we have measured the activation of vimentin, α-SMA and Wnt3 mRNA using TGF-β-stimulated fibroblasts and identified that CHST15 siRNA significantly reduced the mRNA expression levels of these markers (Fig 6D). In addition, we have also measured the effect of CHST15 siRNA on the expression of EMT markers such as E-cadherin, vimentin, BMP7 and Wnt3 using TGF-β-stimulated colon cancer cell line and identified a significant suppression of these markers in the CHST15 siRNA treated cells (Fig 6E).

**Table 1:** The amounts of CS in condition medium of siRNA-treated colon fibroblasts.

	pmol/mg protein (mol%)	
	Negative control siRNA	CHST15 siRNA
ΔHexUA-GalNAc	314 ± 40 (2.6 ± 0.3)	441 ± 47 (3.7 ± 0.4)
ΔHexUA-GalNAc(6S)	2,780 ± 189 (22.8 ± 0.8)	2,640 ± 122 (21.5 ± 0.7)
ΔHexUA-GalNAc(4S)	8,045 ± 189 (66.2 ± 0.9)	8,542 ± 97 (69.8 ± 0.3)
ΔHexUA(2S)-GalNAc(6S)	226 ± 15 (1.9 ± 0.1)	230 ±13 (1.8 ± 0.1)
ΔHexUA(2S)-GalNAc(4S)	128 ± 21 (1.0 ± 0.2)	196 ± 27 (1.6 ± 0.2)
ΔHexUA-GalNAc(4S,6S)	**672 ± 57 (5.5 ± 0.3)**	**196 ± 6 (1.6 ± 0.1)**
Total CS disaccharide	12,165 ± 419 (100)	12,244 ± 173 (100)

In addition, we have performed additional experiments in severe, acute DSS colitis that was induced by combination with compound 48/80 (C48/80). We have discovered that the C48/80 dramatically augmented the CHST15 mRNA expression and thickening of submucosal area in acute DSS model. CHST15 siRNA significantly reduced the DAI, CHST15 mRNA expression, thickening of the submucosa and IL-6 levels (S1 Fig).

## Discussion

In response to tissue injury, the balance between “adequate” and “fibrotic” wound healing is an important factor to successfully recover the damaged tissue. Persistent local inflammation and fibrosis may skew the host defense system towards more “fibrotic” healing instead of “adequate” healing [8–10]. The molecular mechanism dictating the balance has been reported in various wound healing or fibrosis models, but it is not fully explored in chronic, progressive destructive diseases including IBD. Endogenous regeneration-promoting factors like HGF were actually up-regulated in response to injury in IBD patients as well as mouse DSS colitis [19,28], although this alone may not be enough to recover the damaged tissue in case of chronic ulcers with certain inflammatory conditions. Experimentally, forced expression of regeneration-promoting factors or inhibited activation of fibroblasts was shown to promote epithelial healing [19,29], supporting the importance of skewing host response towards “adequate” healing. We hypothesized that CHST15 and its product CS-E play a pivotal role in creating a local fibrotic field around injured sites such as ulcers and investigated the balance between MH response and fibrosis in the colon.

In mouse DSS colitis, accumulation patterns of F4/80^+^ macrophages and ER-TR7^+^ fibroblasts (Fig 1C) suggest the “shift” of main disease condition from inflammation to fibrosis in this model. CHST15 mRNA was inducible and its strong expression was observed during the “shifting” period showing co-existence of ulcer, inflammation and fibrosis. Since the CHST15 mRNA expression level remained higher during active fibrosis phase by day 19 despite decrease in inflammatory macrophages, progressively increased fibroblasts are considered to be involved in CHST15 mRNA expression. CHST15^+^ cells were barely detected in normal colon but increased within mucosal and submucosal mononuclear cells during active fibrosis phases (Fig 5E). In morphology, fibroblast-like cells were main producers of CHST15 protein at fibrosis phase. Although not specific for E-unit, significant deposition of CS chains recognized by antibody CS56 was detected within mucosa and submucosal layers as well (Fig 5E). The signal was not limited to mononuclear cells and detectable in matrix component, but main producer was also judged as fibroblast-like cells. The involvement of inducible CHST15 mRNA, the protein product, and CS in fibrotic colonic injury was demonstrated in this model.

The effect of CHST15 siRNA on MH and fibrosis via systemic route was first tested in chronic phase, characterizing impaired epithelial architecture and enlarged interstitial space with inflammatory infiltrate (Fig 3E). Fibroblasts, but no more macrophages, were the major component of the interstitial inflammatory infiltrate. CHST15 siRNA reduced the accumulation as well as activation of fibroblasts as evidenced by reduced α-SMA mRNA and the encoded protein. Induction of MH by the treatment with CHST15 siRNA was evident as epithelial architecture showing almost normal morphology compared to control. Induction of MH response was also supported by rapid reduction of fecal blood by CHST15 siRNA. The results indicate that CHST15 siRNA skews host defense towards MH instead of fibrosis in chronic fibrosis phase.

To translate CHST15 siRNA into clinical practice, we established submucosal injection, which allows naked siRNA to remain within the colon but to be degraded immediately after entering into circulation. The ability of siRNA diffusion within the submucosal space in a pancolonic and circumferential fashion was more pronounced as expected and almost the entire colon could be targeted by this approach in mice (Fig 4). Submucosally delivered siRNA was detectable at least 6 days after injection with unbound form, suggesting higher stability within the matrix space. Fluorescence-signal was actually detectable in both fiber-like components and cells that contact the fiber, suggesting that siRNA is first trapped by matrix component and subsequently incorporated with neighboring cells. The colonic submucosa is thus characterized as having “nature’s reservoir”, enabling naked siRNA to function at sites of local injury with a minimum risk of systemic side effects. With this clinically applicable approach, the effect of CHST15 siRNA on chronic colitis was tested via submucosal route and with clinically-relevant parameters (Fig 5). Endoscopic submucosal injection of CHST15 siRNA actually reduced CHST15 mRNA, CHST15 protein, CS and collagen deposition, indicating its anti-fibrotic property. Induction of MH by submucosally injected CHST15 siRNA was endoscopically and histologically evident. Endoscopic submucosal injection is therefore effective for the delivery of CHST15 siRNA to achieve MH at sites of fibrotic colonic injury.

Considering the effect of CHST15 siRNA on the reduced accumulation of fibroblasts, a concern raised that it inhibits wound healing since fibroblasts are also recruited to reseal the wound at early phase after tissue injury. To examine the possibility, the effect of CHST15 siRNA via systemic route was tested on acute phase characterized by mucosal defects with inflammatory infiltrate (Fig 2). CHST15 siRNA significantly reduced the accumulation of fibroblasts as found in chronic colitis. However, the degree of reduction was not under the normal level and the epithelial morphology was maintained, suggesting that CHST15 siRNA acts on newly increased fibroblasts without affecting epithelial resealing. Instead, CHST15 siRNA promoted MH as supported by reduction of fecal blood and increased Ki-67^+^ epithelial cells. In addition, CHST15 siRNA reduced serum IL-6 level and the excessive accumulation of macrophages that was distinct from chronic colitis. Thus, CHST15 siRNA skews host defense towards MH induction additionally with anti-inflammatory property *in vivo* in acute tissue injury.

Colonic fibroblasts were tested *in vitro* to identify whether CHST15 siRNA-mediated reduction of activated fibroblasts impacts on inflammation, MH or other mechanisms (Fig 6). Significant reduction of IL-6 by CHST15 siRNA may result, at least in part, in the anti-inflammatory activity during acute DSS colitis. TGF-β significantly increased the mesenchymal marker vimentin, indicating the phenotype change to mesenchymal cells. CHST15 siRNA significantly reduced the expression levels of CHST15 and vimentin (Fig 6), indicating the inhibition of EMT phenotype change. In addition, CHST15 siRNA augmented BMP-7 and to a lesser extent, E-cadherin (Fig 6), also supporting the notion that CHST15 siRNA inhibited the EMT change [30, 31]. Thus, CHST15 siRNA reduced EMT signaling but increased EMT-reversing properties by colon cancer cells. Although further investigations are needed, the simultaneous effects of both MH-induction and fibrosis-suppression found in acute and chronic colitis are attributable in part to EMT-reversing action on fibroblasts by CHST15 siRNA.

## Conclusion

In summary, CHST15 is augmented at the site of colon injury. CHST15 siRNA reduced the expression of CHST15 mRNA and protein in the colon and the area of fibrosis *in vivo*. Blockade of CHST15 inhibited the activation of colon fibroblasts and reversed the EMT changes by colon cancer cells *in vitro*. Together with effective pancolonic delivery, endoscopic local injection of CHST15 siRNA would provide a novel therapeutic option for local refractory lesions having histological fibrosis in IBD.

## Supporting Information

S1 Fig Reduced inflammation by systemic CHST15 siRNA in mouse acute colitis. **(A)** Experimental design. Compound 48/80 (C48/80; MP Biomedicals) was injected intraperitoneally with a dose of 1 mg/100 mL PBS per mouse at day 1 of DSS. Negative control siRNA or CHST15 siRNA was injected intraperitoneally at day 2, and then the mice were sacrificed at day 5. (**B**) Disease activity index (DAI). (**C**) Effect of CHST15 siRNA on the expressions of CHST15 mRNAs at day 5. Statistical analyses using Student’s t-test are shown; negative control siRNA (DSS + control siRNA) vs. CHST15 siRNA (DSS + CHST15 siRNA) in DSS-treated groups and negative control siRNA (DSS + control siRNA) vs. CHST15 siRNA (DSS + CHST15 siRNA) in DSS + C48/80-treated groups. (**D**) Representative Masson’s Trichrome staining of the colon at day 5. Original magnifications, x100. (**E**) Effect of CHST15 siRNA on serum IL-6. Results are expressed as mean ± SD (n = 5). *p<0.05, **p<0.01 and ***p<0.001 vs. corresponding negative control siRNA treatment group by Student’s t-test. Representative data were shown from 2 independent experiments.
(TIFF)

S1 File Supplementary methods. Effect of CHST15 siRNA treatment on a severe colitis model induced by compound 48/80 and DSS in mice.
(DOC)
