# Supplementary figures and images for: Pivotal Role of Carbohydrate Sulfotransferase 15 in Fibrosis and Mucosal Healing in Mouse Colitis
Source: PLoS One. 2016 Jul 13;11(7):e0158967. doi: 10.1371/journal.pone.0158967 (PMC4943596; doi:10.1371/journal.pone.0158967)

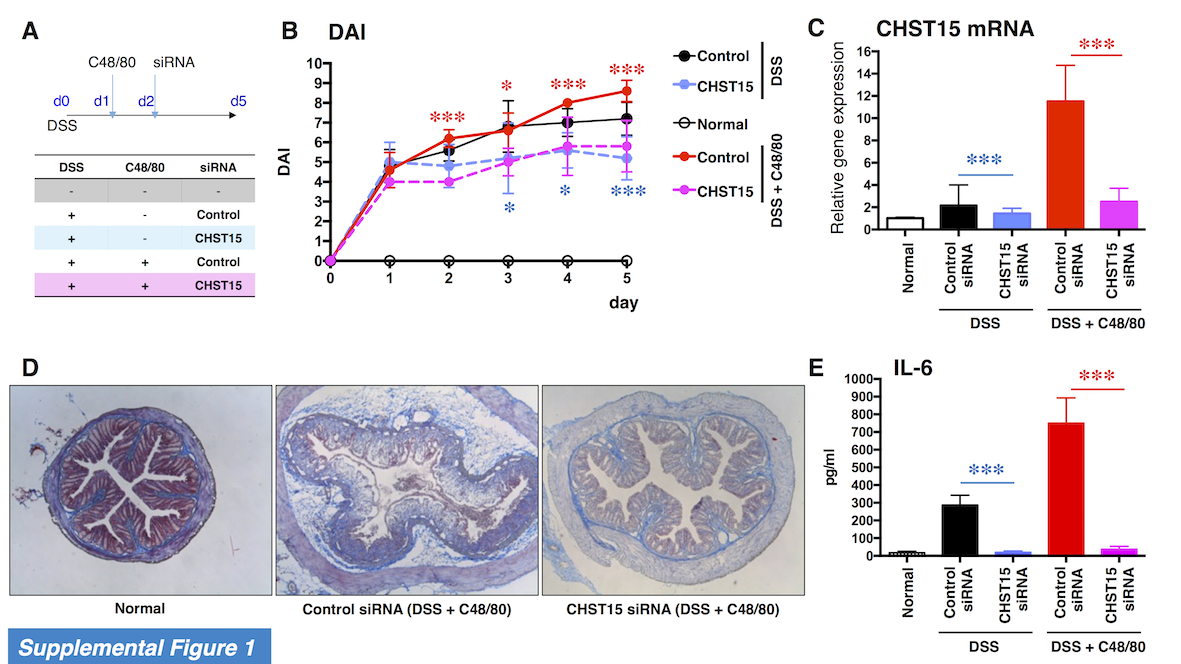

Supplement: S1 Fig — (A) Experimental design. Compound 48/80 (C48/80; MP Biomedicals) was injected intraperitoneally with a dose of 1 mg/100 mL PBS per mouse at day 1 of DSS. Negative control siRNA or CHST15 siRNA was injected intraperitoneally at day 2, and then the mice were sacrificed at day 5. (B) Disease activity index (DAI). (C) Effect of CHST15 siRNA on the expressions of CHST15 mRNAs at day 5. Statistical analyses using Student’s t-test are shown; negative control siRNA (DSS + control siRNA) vs. CHST15 siRNA (DSS + CHST15 siRNA) in DSS-treated groups and negative control siRNA (DSS + control siRNA) vs. CHST15 siRNA (DSS + CHST15 siRNA) in DSS + C48/80-treated groups. (D) Representative Masson’s Trichrome staining of the colon at day 5. Original magnifications, x100. (E) Effect of CHST15 siRNA on serum IL-6. Results are expressed as mean ± SD (n = 5). *p<0.05, **p<0.01 and ***p<0.001 vs. corresponding negative control siRNA treatment group by Student’s t-test. Representative data were shown from 2 independent experiments. (TIFF) [file pone.0158967.s001.tiff]
