# Supplementary material for: Pivotal Role of Carbohydrate Sulfotransferase 15 in Fibrosis and Mucosal Healing in Mouse Colitis
Source: PLoS One. 2016 Jul 13;11(7):e0158967. doi: 10.1371/journal.pone.0158967 (PMC4943596; doi:10.1371/journal.pone.0158967)
Supplement: S1 File — Effect of CHST15 siRNA treatment on a severe colitis model induced by compound 48/80 and DSS in mice. (DOC) [file pone.0158967.s002.doc]

**Supplementary method**

Compound 48/80 (C48/80; MP Biomedicals) was injected intraperitoneally with a dose of 1 μg/100 μL PBS per mouse at day 1 of DSS. Negative control siRNA or CHST15 siRNA was injected intraperitoneally at day 2, and then the mice were sacrificed at day 5. The disease activity index (DAI) was calculated. The expression level of CHST15 mRNA at day 5 was also estimated. Masson’s Trichrome staining was done to identify the colon fibrosis and the anti-fibrotic effect of CHST15 siRNA at day 5. Finally, the effect of CHST15 siRNA on serum IL-6 was also estimated. Statistical analyses were performed using Student’s t-test.

**Figure legends**

**S1 Fig.** **Reduced inflammation by systemic CHST15 siRNA in mouse acute colitis**.

1. Experimental design. Compound 48/80 (C48/80; MP Biomedicals) was injected intraperitoneally with a dose of 1 μg/100 μL PBS per mouse at day 1 of DSS. Negative control siRNA or CHST15 siRNA was injected intraperitoneally at day 2, and then the mice were sacrificed at day 5. (**B**) Disease activity index (DAI). (**C**) Effect of CHST15 siRNA on the expressions of CHST15 mRNAs at day 5. Statistical analyses using Student’s t-test are shown; negative control siRNA (DSS + control siRNA) vs. CHST15 siRNA (DSS + CHST15 siRNA) in DSS-treated groups and negative control siRNA (DSS + control siRNA) vs. CHST15 siRNA (DSS + CHST15 siRNA) in DSS + C48/80-treated groups. (**D**) Representative Masson’s Trichrome staining of the colon at day 5. Original magnifications, x100. (**E**) Effect of CHST15 siRNA on serum IL-6. Results are expressed as mean ± SD (n=5). *p<0.05, **p<0.01 and ***p<0.001 vs. corresponding negative control siRNA treatment group by Student’s t-test. Representative data were shown from 2 independent experiments.
